# The characteristics and related factors of insomnia among postoperative patients with gastric cancer: a cross-sectional survey

**DOI:** 10.1007/s00520-021-06295-6

**Published:** 2021-05-27

**Authors:** Guang-hui Zhu, Juan Li, Jie Li, Bo-wen Xu, He-ping Wang, Xin-miao Wang, Jia-qi Hu, Ming-hao Dai

**Affiliations:** 1 Guang’anmen Hospital, China Academy of Chinese Medical Sciences, No. 5, Beixian Ge Street, Xicheng District, Beijing, 100053 China; 2 Graduate School, Beijing University of Chinese Medicine, No. 11, Beisanhuan Dong Road, Chaoyang District, Beijing, 100029 China; 3 Peking University Health Science Center, No. 38, Xueyuan Road, Haidian District, Beijing, 100191 China

**Keywords:** Gastric cancer, Insomnia, Postoperative, Characteristics, Related factors

## Abstract

**Purpose:**

This study aims to explore the characteristics and related factors of insomnia of patients after operation for gastric cancer.

**Methods:**

A cross-sectional survey was carried out and finally 115 patients with insomnia after operation for gastric cancer were included. The general information, gastric cancer-related information, sleep quality, and other symptoms were investigated.

**Results:**

① The Pittsburgh sleep quality index score of most insomnia patients after gastric cancer surgery was 11-15 points, and the sleep quality rating was “poor”. ② The sleep quality of patients with insomnia after surgery for gastric cancer is related to the number of chemotherapy cycles, fatigue, and depression. ③ The probability of reduced sleep quality with the number of chemotherapy cycles >6 is 3.640 times that of ≤6. The probability of reduced sleep quality during moderate to severe fatigue was 4.390 times that of patients with no or mild fatigue.

**Conclusion:**

Attention to related factors may be associated with improvement of sleep quality in patients with gastric cancer after surgery.

**Supplementary Information:**

The online version contains supplementary material available at 10.1007/s00520-021-06295-6.

## Introduction

According to the World Health Organization (WHO), there were 896.63 million cancer deaths worldwide in 2016, making it the second leading cause of death [1]. Based on GLOBOCAN 2018 statistics, the incidence and death of gastric cancer accounted for the 5th and 3rd of all malignant tumors all over the world, respectively [2]. Anti-tumor tends to be comprehensive measures [3–5]. With the continuous improvement of treatment, the survival period of cancer patients has been prolonged. Improving the quality of life of cancer patients has gradually become the focus of attention and urgent problems to be solved for patients, families, society, and clinicians. Symptom management of tumor patients has gradually become a hot spot for medical personnel and scientific researchers [6, 7].

Insomnia can lead to adverse health problems. In addition to the potential for physical, psychological, and social problems, they are more likely to contribute to the development of diseases, including cardiovascular disease and cancer [8]. Patients with insomnia have a moderate risk of developing cancer [8–11]. Studies have shown that insomnia damages DNA and reduces the body’s ability to repair it, possibly increasing the risk of cancer [12]. In addition, short sleep duration has been linked to cancer mortality [13]. The incidence of insomnia in patients with malignant tumors is 52.6-67.4%, which is twice that of the general population [14, 15]. However, if effective intervention is not given, chronic insomnia often develops [16, 17]. It will increase the psychological burden of cancer patients, reduce the effect of anti-tumor treatment, and seriously affect the health of patients [18, 19]. Researches, in recent years, have found that insomnia became a common clinical symptom in cancer patients, but patients and doctors did not pay enough attention and treatment to insomnia [17, 20]. Early intervention may reduce the severity or longevity of these problems during treatment [21].

In 1986, Spielman et al. have introduced the behavioral perspective on insomnia (the “3P” model), and conceptualized the influencing factors of insomnia into three categories: predisposing factors for insomnia, precipitating factors for insomnia attacks, and perpetuating factors for maintaining long-term insomnia [22]. At present, the “3P” model is still a popular model for the pathogenesis of chronic insomnia [23]. This model believes that individual susceptibility and innate tendency (such as high arousal or anxiety), and encounter predisposing factors (such as stressful events or diseases), will lead to acute insomnia. However, negative thoughts and non-adaptive coping behaviors (such as excessive concern and worry about insomnia) will produce conditioned arousal, and chronic insomnia will occur after repeated [24]. Most studies have included small sample sizes of participants with different characteristics, which may make it impossible to conduct subgroup analysis and identify potential related factors for insomnia [25]. Although a number of factors have been linked to the development of insomnia in cancer patients (e.g., gender, age, cancer treatment), the results have been mixed when it comes to related factors [26]. Surgical resection is the preferred treatment for gastric cancer [27]. Wang X. et al. found that the incidence of disturbed sleep was 90.48% and the risk of symptoms was about 4 points through investigation and analysis of the symptoms of patients after gastric cancer [28]. However, no studies have explored the characteristics and influencing factors of insomnia in postoperative patients with gastric cancer.

Recent studies have shown that insomnia is linked to other symptoms. The simultaneous occurrence of at least two symptoms can be classified into clusters of symptoms that are clinically significant and interrelated [29, 30]. Different clusters of sleep-related symptoms, including fatigue and depression, have been reported in cancer patients [31–33]. However, due to the limitations of previous studies, the association between insomnia and other symptoms has not been fully studied, including the lack of effective questionnaires to measure insomnia [31–35].

Based on the “3P” model of insomnia, we started this study in order to understand the characteristics of postoperative insomnia in patients with gastric cancer, find out the possible related factors for insomnia, and explore the interaction between insomnia and other symptoms.

## Methods

### Participants

The cross-sectional study included patients who attended the Oncology Clinic of Guang’anmen Hospital, China Academy of Chinese Medical Sciences between April 2019 and January 2020. The diagnosis of gastric cancer refers to “Regulations for Diagnosis and Treatment of Gastric Cancer (2018 Edition)” [3] and “American Joint Committee on Cancer (AJCC) gastric cancer TNM staging (the 8th version)” [36]. Insomnia is diagnosed on the “Chinese Classification and Diagnostic Criteria for Mental Disorders” (the 3rd version) [37]: ① difficulty falling asleep, ② often awakening and unstable sleep, ③ difficulty falling asleep again after waking up, awakening prematurely in the morning, ④ sleeping less than 6 h, lack of energy during the day, and drowsiness, and ⑤ history of repeated attacks, occurring ≥3 times a week and lasting ≥1 month.

The cases were screened according to the following: ① The patients diagnosed with gastric cancer, and the staging being T_1-4_N_X_M_0_; ② within 6-8 months after standard radical gastrectomy; ③ meeting the diagnostic criteria for insomnia, and the Pittsburgh sleep quality index (PSQI) score being > 5 points; ④ no symptoms of insomnia within 1 month before surgery and not taking drugs with sleeping effects (based on clinical records or as reported by patient); ⑤ over 18 years old; ⑥ Karnofsky (KPS) score being ≥ 60 points; ⑦ having sufficient cognitive ability to complete the survey; and ⑧ signing the informed consent form voluntarily.

### Procedures

This study was part of a project of Beijing Science and Technology Commission. The proposal was approved by the Ethics Committee of Guang’anmen Hospital, China Academy of Chinese Medical Sciences (NO. 2016-118-KY-02). Potential participants were approached and invited to the study on the first day they were admitted to the hospital. This was a convenience sample. The method and purpose of the research were explained to them. After the patients signing the informed consent form, the inclusion criteria were determined, and information was collected for patients who met the inclusion criteria.

### Measurements

(1) General information includes age, gender, and KPS score; (2) relevant data of gastric cancer are pathological type, differentiation grade, Lauren grade, clinical staging, TNM staging, lesion location, surgical method, and number of chemotherapy cycles; (3) main observation index is PSQI scale score; (4) other observation indicators are Piper Fatigue Scale-Chinese Version (PFS-CV) score, and Hospital Anxiety and Depression Scale (HADS) score.

#### PSQI

The PSQI scale is used to evaluate sleep quality. It consists of self-evaluation and other-evaluated items, and only 19 self-evaluation items participated in the scoring, including 7 components [38]. Every component is scored on a scale of 0-3, and the total score ranges from 0 to 21. PSQI score = component (A + B + C + D + E + F + G). Table 1 presents the correlation between total PSQI score and sleep quality. The score is higher, the worse the quality of sleep. The diagnostic sensitivity of this scale is 89.6%, and the specificity is 86.5% (kappa = 0.75, P ≤ 0.001) [21].

**Table 1:** The correlation between total PSQI score and sleep quality

The total score of PSQI	Sleep quality
0-5	Good
6-10	General
11-15	Poor
16-21	Very poor

#### PFS-CV

The PFS-CV was translated by scholars from Hong Kong in 2003, including 4 dimensions of behavior, emotion, feeling, and cognition, with a total of 22 contents. The repeat test reliability is 0.98 [39]. Every content contains 11 points (0-10 points). Patients are scored according to the degree of their fatigue. Among them, there are 5 items for evaluating feelings and emotions, and 6 items for evaluating cognition and behaviors. The final score is derived from the average score of 4 dimensions. The score is higher, the heavier the fatigue; 0 means asymptomatic, 1-3 means mild, 4-6 means moderate, and 7-10 means severe.

#### HADS

In 1983, Zigmond AS. et al. designed HADS [40]. After tested, the anxiety subscale (A), depression subscale (D), and HADS total score have good retest consistency [41]. There are a total of 14 questions in the table. Among them, the seven questions of A test are used to evaluate anxiety, and the seven questions of D test are used to evaluate depression. Every question is counted as 0-3 points according to the degree level. The score is higher, the more severe the degree; 0-7 is asymptomatic, 8-10 is suspicious, and 11-21 is symptomatic. It is divided into 8 boundaries, <8 is negative, and ≥ 8 is positive.

### Data analysis

① Statistical description method includes mean ± standard deviation (‾x ± S), composition ratio, and median and quartile [M (Q25, Q75)].

② Statistical inference method: When the data conforms to the normal distribution and the variance is uniform, the independent sample t test is used to compare the samples between groups. When the variance is not uniform, the independent sample approximate t test is used. Non-normally distributed samples between groups use non-parametric test (Mann-Whitney U test). One-way ANOVA test or Kruskal-Wallis test was used for sample comparison among multiple groups. Multivariate binary unconditional logistic regression analysis is used to analyze categorical variables. Pearson correlation coefficient is used for correlation analysis of numerical variables.

The SPSS 24.0 software was used for statistical analysis of all data, using a two-sided difference test. P ≤ 0.05 is considered statistically significant.

## Results

**Fig. 1:**
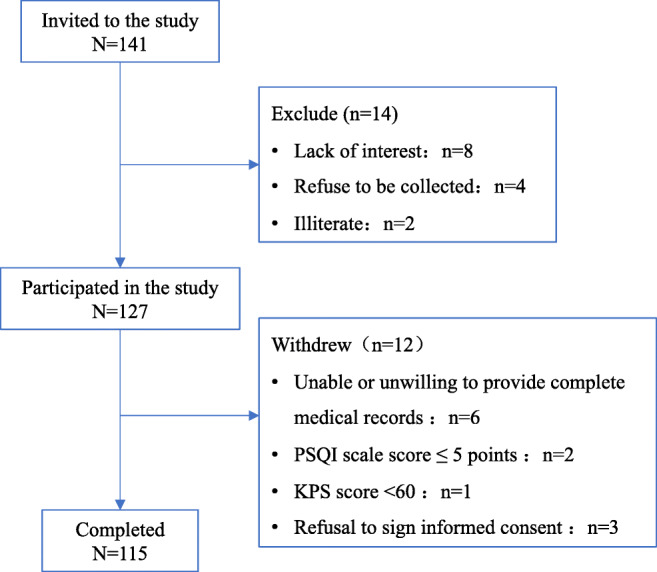
Flowchart of the study recruitment

A total of 141 cases of postoperative patients with gastric cancer were initially screened, and 115 cases were finally included. According to the rough estimation method of sample size, the sample size is 5-10 times the number of variables [42]. The number of variables in this study is 15, requiring a sample size of 75-150 cases, so the number of cases included in this study met the requirements. Figure 1 presents the process of recruiting study participants. General data, gastric cancer data, and other symptom data of the included cases were presented in Table 2.

**Table 2:** Basic information of included cases (n = 115)

Group	Number	Percent
Gender group		
Male	73	63.48%
Female	42	36.52%
Age group		
≤60	63	53.78%
>60	52	46.22%
KPS group		
<90	35	30.43%
≥90	80	69.57%
Lesion site		
Gastric cardia	22	21.36%
The body of the stomach	38	36.89%
The antrum of the stomach	43	41.75%
Degree of differentiation		
High differentiation	4	3.81%
Poor differentiation	60	57.14%
Moderate differentiation	28	26.67%
Moderate to poor differentiation	13	12.38%
Lauren type		
Intestinal type	26	28.89%
Diffuse type (stomach type)	44	48.89%
Hybrid	20	22.22%
Clinical stage		
I	6	5.22%
II	41	35.65%
III	68	59.13%
T stage		
T1	7	6.09%
T2	26	22.61%
T3	48	41.74%
T4	34	29.56%
N stage		
N0	24	20.87%
N1	34	29.57%
N2	25	21.74%
N3	32	27.83%
Surgical methods		
Proximal gastrectomy	19	16.52%
Distal gastrectomy	38	33.04%
Total gastrectomy	58	50.43%
The number of chemotherapy cycle		
≤6	55	52.88%
>6	49	47.12%
PSQI score		
7-10	37	32.17%
11-15	60	52.17%
16-21	18	15.65%
PFS-CV score		
≤4	43	37.39%
>4	72	62.61%
HADS score		
Anxiety subtable		
<8	17	14.78%
≥8	98	85.22%
Depression subtable		
<8	28	24.35%
≥8	87	75.65%

### The characteristics

There were 60 cases (52.17%) with a PSQI score of 11-15, 37 cases (32.17%) with a score of 7-10, and 18 cases (15.65%) with a score of 16-21. The average score of the PSQI score was 12.05 ± 3.25. Among them, the “daytime dysfunction” component had the highest score, and the hypnotic drug component had the lowest score (see Table 3).

**Table 3:** PSQI scale components and scores

	Score (‾x ± S)
A. Sleep quality	2.09 ± 0.78
B. Time to fall asleep	2.04 ± 0.73
C. Sleep time	1.90 ± 0.82
D. Sleep efficiency	1.68 ± 1.01
E. Sleep disorders	1.45 ± 0.63
F. Hypnotic drugs	0.63 ± 1.12
G. Daytime dysfunction	2.17 ± 0.85
Total score	11.96 ± 3.31

### Related factors

#### General information

The independent sample t test was used to compare the PSQI scores of gender and age groups, t = −0.010 and −1.092, *P* all >0.05. The PSQI score of KPS group was subjected to non-parametric test (Mann-Whitney U test), Z = −0.010, *P* > 0.05; Table 4.

**Table 4:** Correlation analysis of general data and PSQI score

		n	PSQI score (‾x ± S)	t	*P*
Gender	Male	73	11.96 ± 3.08	−0.010	0.992^▲^
	Female	42	11.95 ± 3.71		
Age	≤60	63	11.65 ± 3.66	−1.092	0.277^▲^
	>60	52	12.33 ± 2.81		
			M (Q25, Q75)	Z	*P*
KPS	<90	18	12.00 (12.00, 14.00)	−1.706	0.088^▲^
	≥90	40	11.00 (8.25, 14.00)		

^▲^*P* > 0.05

#### Relevant data of gastric cancer

Kruskal-Wallis tests were respectively performed on the PSQI scores among the groups of the lesion site, differentiation grade, Lauren type, clinical stage, N stage, and surgical method, χ^2^ = 3.111, 4.664, 2.350, 1.576, 0.715, and 5.292, *P* all >0.05. One-way ANOVA test was used in the PSQI scores among the group of the T stage, F = 0.793, *P* > 0.05; Table 5.

**Table 5:** Correlation analysis between gastric cancer data and PSQI score

		n	PSQI score M (Q25, Q75)	χ^2^	*P*
Lesion site	Gastric cardia	22	11 (8, 12)	3.111	0.211^▲^
	Body of the stomach	38	12.5 (8, 16)		
	Antrum of the stomach	43	12 (11, 14)		
Differentiation grade	High differentiation	4	12 (12, 12)	4.664	0.198^▲^
	Poor differentiation	60	11 (9, 13.75)		
	Moderate differentiation	28	13.5 (8, 16)		
	Moderate to poor differentiation	13	11 (8, 12.5)		
Lauren type	Intestinal type	26	12 (9.75, 16.25)	2.350	0.309^▲^
	Diffuse type (stomach type)	44	12 (10, 14)		
	Hybrid	20	11 (8, 13)		
Clinical stage	I	6	13 (11, 16)	1.576	0.455^▲^
	II	41	12 (9, 13.5)		
	III	68	12 (10, 14)		
N stage	N_0_	24	12 (10.25, 15.25)	0.715	0.870^▲^
	N_1_	34	12 (9, 14)		
	N_2_	25	12 (9.5, 14)		
	N_3_	32	12 (10, 14.75)		
Surgical methods	Proximal gastrectomy	19	11 (7, 12)	5.292	0.071^▲^
	Distal gastrectomy	38	12 (11, 14)		
	Total gastrectomy	58	12 (8.75, 14.25)		
		n	(‾x ± S)	F	*P*
T stage	T_1_	4	12.25 ± 0.96	0.793	0.503^▲^
	T_2_	13	12.92 ± 3.82		
	T_3_	24	12.21 ± 3.40		
	T_4_	17	11.12 ± 2.89		

^▲^*P* > 0.05

The PSQI scores of the number of chemotherapy cycles grouped (≤6 cycles, >6 cycles) were subjected to non-parametric test (Mann-Whitney U test), Z = −4.447, *P* = 0.000; Supplementary Table 1.

#### Fatigue, depression, and anxiety

The Pearson correlation coefficients between the total PSQI score and the final score of the PFS-CV scale, between the total PSQI score and the HADS scale (depression subscale) score, and between the total PSQI score and the HADS scale (anxiety subscale) score were respectively 0.428, 0.261, and 0.060. *P* = 0.000, 0.005, and 0.527; Supplementary Figure 1, Supplementary Figure 2, and Supplementary Figure 3.

### Related factors

The PSQI score was used as the dependent variable and assigned a value: 0 = “PSQI score <11 points (sleep quality is general)” and 1 = “PSQI score ≥11 points” (sleep quality is poor or very poor). The above related factors with *P* < 0.05 (the number of cycles of chemotherapy, the PFS-CV scale, the HADS scale (depression subscale)) were used as independent variables and assigned values: 0 = “the number of chemotherapy cycles ≤6,” 1 = “the number of chemotherapy cycles >6”; 0 = “PFS-CV scale ≤4 points,” 1 = “PFS-CV scale >4 points”; and 0 = “HADS scale (depression subscale) <8 points,” 1 = “HADS scale (depression subscale) ≥ 8 points”. A multivariate binary unconditional logistic regression model was established, and the forward method was used to select and eliminate independent variables. The regression analysis showed that the number of chemotherapy cycles and fatigue was significant related factors for the reduction of sleep quality in patients with insomnia (*P* < 0.05).

Compared with the number of chemotherapy cycles ≤ 6, when the number of previous chemotherapy cycles after gastric cancer surgery is greater than 6, the risk of reduced sleep quality is increased (OR = 3.640, 95% CI: 1.416-9.357, *P* = 0.007). Compared with patients with no or mild fatigue, patients with moderate or severe fatigue after gastric cancer surgery have an increased risk of sleep quality (OR = 4.390, 95% CI:1.843-10.460, *P* = 0.001; Supplementary Table 2).

## Discussion

This study investigated the characteristics of sleep quality included in patients. It was found that poor sleep quality accounted for 52.17%; patients with very poor sleep quality accounted for 15.65%. In the analysis of related factors, the total PSQI score was not statistically different among the groups of gender, age, KPS score, gastric cancer lesion location, differentiation grade, Lauren classification, clinical stage, T stage, and N stage (*P* > 0.05). That showed that the severity of insomnia in patients after gastric cancer surgery was not significantly related to the above factors. At present, some studies have concluded that gender, age, cancer stage, and other factors have nothing to do with the degree of insomnia in the included cases, which was the same as the results of this study [20, 21, 43]. However, there were still studies that differ from the results of this study. Ohayon MM et al. believed that gender and age were factors that induce insomnia in cancer patients [44]. We will need to design a prospective study including a larger sample size to verify the conclusions.

By analyzing the influence of the number of chemotherapy cycles on the degree of insomnia, it was found that the total PSQI score of patients with chemotherapy cycles > 6 [13 (12, 16)] was significantly higher than that of patients with chemotherapy cycles ≤ 6 [10 (8, 12)] (P = 0.000). The multivariate binary unconditional logistic regression analysis showed that the number of chemotherapy cycles > 6 was a significant related factor for reduced sleep quality in patients with insomnia after operation for gastric cancer (*P* < 0.05). The risk of reduced sleep quality with the number of chemotherapy cycles >6 is 3.640 times that of ≤6. Chemotherapy, as one of the main anti-tumor treatments, has a certain cytotoxic effect, causing patients to have side effects such as anorexia, nausea, vomiting, fatigue, pain, and bone marrow suppression. As a result, the patient loses confidence in the treatment effect, and insomnia also occurs or worsens [45, 46]. The result of a cross-sectional study looking at new-onset insomnia in cancer patients during chemotherapy showed that 42.8% of the 213 patients surveyed reported insomnia, and 31.9% of them reported severe insomnia [6]. In addition, the cerebral toxicity caused by the use of chemotherapeutic drugs contributes to the progression of brain inflammation by affecting the production and distribution of pro-inflammatory cytokines [47]. With the prolonged use of chemotherapeutic drugs, the tendency of insomnia in patients was aggravated. An 18-month study found that insomnia in cancer patients receiving chemotherapy increased with the length of time they took the drug [48].

The sleep quality of insomnia patients after gastric cancer surgery was positively correlated with fatigue and depression (Pearson = 0.428 and 0.261, *P* = 0.000 and 0.005), but not correlated with anxiety (*P* = 0.527). Hoang HTX et al. also found that the incidence and severity of insomnia in cancer patients had nothing to do with the characteristics of the participants, cancer information, or treatment factors, but were related to the emotional score of the participants [6]. The results of a systematic review also pointed out that patients with insomnia and mood have a two-way effect [49]. The hypothalamus, hippocampus, and other brain tissues regulate emotion and sleep at the same time by secreting neuropeptides and neurotransmitters, so that the two are connected [50, 51]. A Chinese study also found that when a patient suffered from depression and insomnia, the level of neuropeptide Y in the body was lower than that of healthy people, and the level of substance P was higher than that of healthy people [52]. In addition, insomnia and fatigue often occur simultaneously in cancer patients. Xu JN’s study included 60 patients with cancer-related fatigue and found that 70% of them suffered from insomnia [53]. Moreover, this study established a multivariate binary unconditional logistic regression analysis to show that the risk of reduced sleep quality in patients with moderate or severe fatigue after gastric cancer surgery was 4.390 times that of patients with no or mild fatigue (*P* = 0.001).

The advantage of this study was to specifically select patients with insomnia after operation for gastric cancer to explore their sleep quality characteristics and related factors.

### Study limitations

However, this study was a cross-sectional study. There is no control group to give the finding perspective or relevance for the findings of prevalence and severity. Due to its inherent limitations, this study cannot point out how insomnia and its related symptoms affect each other before they appear and how they change throughout the course of the disease. In addition, the insomnia of the included patients in this study was judged based on the history collection and the evaluation of the PSQI, but it may be caused by the error of patients’ recollection that caused the wrong judgment of insomnia. Thirdly, due to limited research funds and time, this study did not observe other symptoms of patients, such as postoperative pain, sleep apnea, and dyspnea, which is a defect of this study. Finally, this study was only a single-center study, so there was certain selection error.

### Clinical implications

This study confirmed that the sleep quality of insomnia patients after operation for gastric cancer was mainly “poor”. That was correlated with the number of chemotherapy cycles, depression, and fatigue. The number of chemotherapy cycles >6 and moderate to severe fatigue was the negative correlation factors for reducing sleep quality in insomnia patients after operation for gastric cancer. Conclusions indicated that the quality of sleep in cancer patients might be related to previous treatments. Early assessment of insomnia and immediate intervention will be needed to improve the quality of life of cancer patients and their treatment compliance. Because insomnia may occur in all stages of cancer development and treatment, some patients have insomnia symptoms in the early stage of the tumor. In the course of treatment, early interventions may reduce the severity or longevity of these problems, such as exercise therapy, psychotherapy, or medication, to prevent the insomnia from worsening with anti-tumor therapy [21, 54]. Cancer patients may have symptom clusters including insomnia, depression, and fatigue, and they may affect each other. Therefore, further research can be carried out to explore the incidence and characteristics of insomnia in cancer patients at different treatment stages.

## Conclusion

Attention to related factors may be associated with improvement of sleep quality in patients with gastric cancer after surgery.

## Supplementary information

ESM 1 Scatter plot of the correlation between sleep quality and fatigue (PNG 15953 kb)

High Resolution Image (TIF 919 kb)

ESM 2 Scatter plot of the correlation between sleep quality and depression (PNG 15953 kb)

High Resolution Image (TIF 919 kb)

ESM 3 Scatter plot of the correlation between sleep quality and anxiety (PNG 15953 kb)

High Resolution Image (TIF 919 kb)

ESM 4 Correlation analysis between chemotherapy cycle and PSQI score (DOCX 14 kb)

ESM 5 Risk factors (DOCX 14 kb)

## Data Availability

Not applicable.
