# Supplementary figures and images for: The characteristics and related factors of insomnia among postoperative patients with gastric cancer: a cross-sectional survey
Source: Support Care Cancer. 2021 May 27;29(12):7315–22. doi: 10.1007/s00520-021-06295-6 (PMC8550093; doi:10.1007/s00520-021-06295-6)

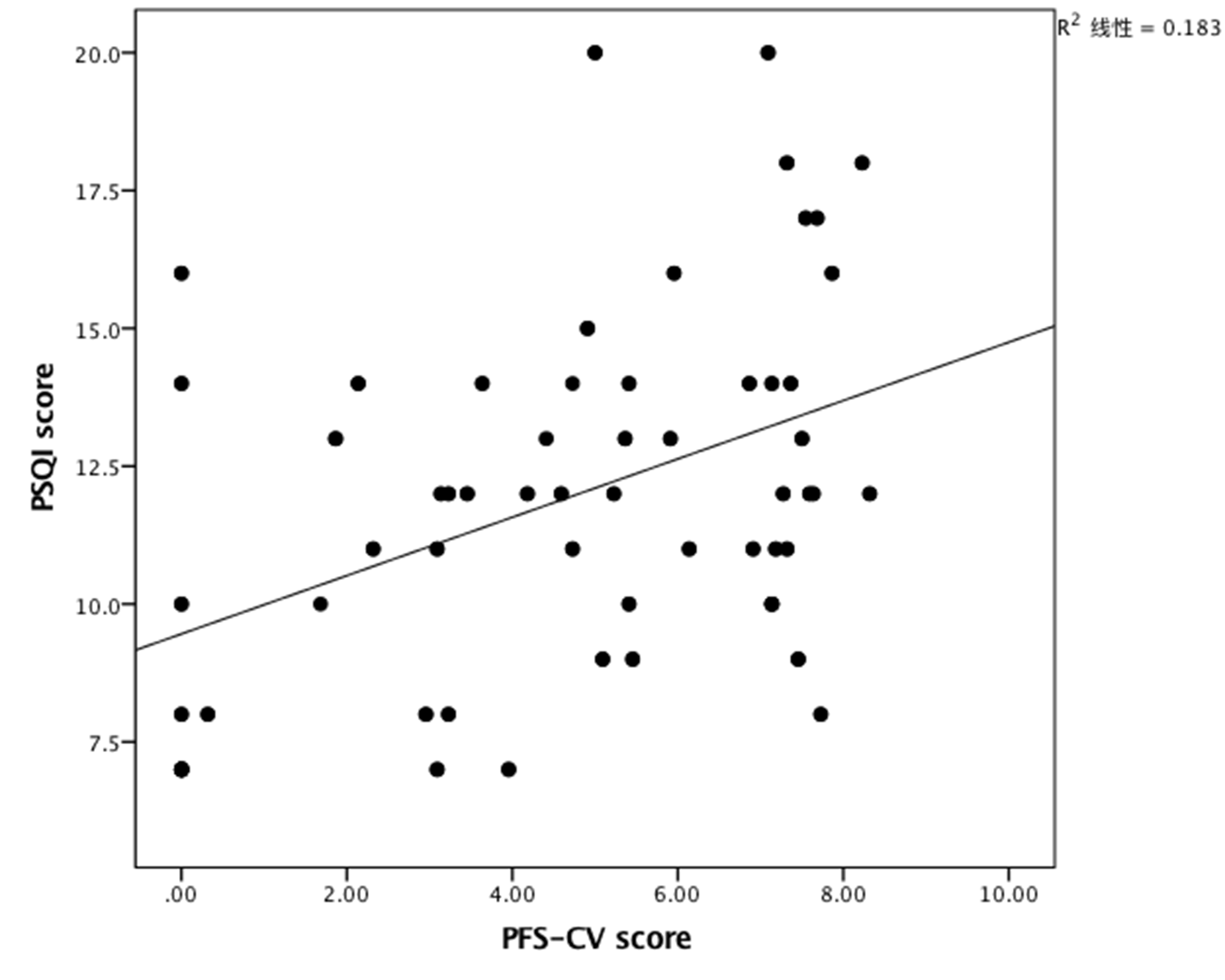

Supplement: Supplementary file 1 — Scatter plot of the correlation between sleep quality and fatigue (PNG 15953 kb) [file 520_2021_6295_Fig2_ESM.png]

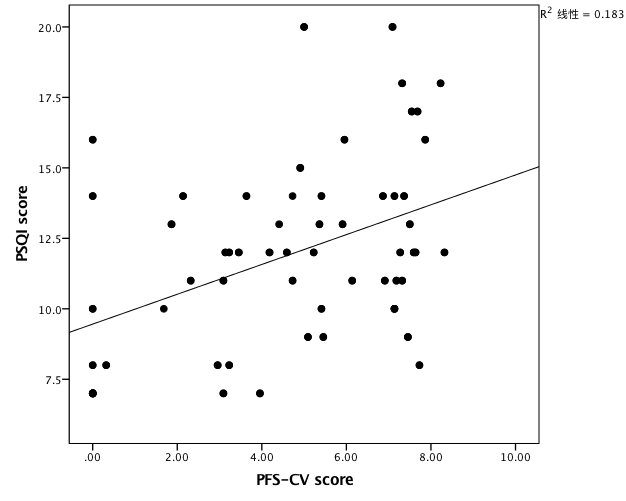

Supplement: Supplementary file 2 — High Resolution Image (TIF 919 kb) [file 520_2021_6295_MOESM1_ESM.tif]

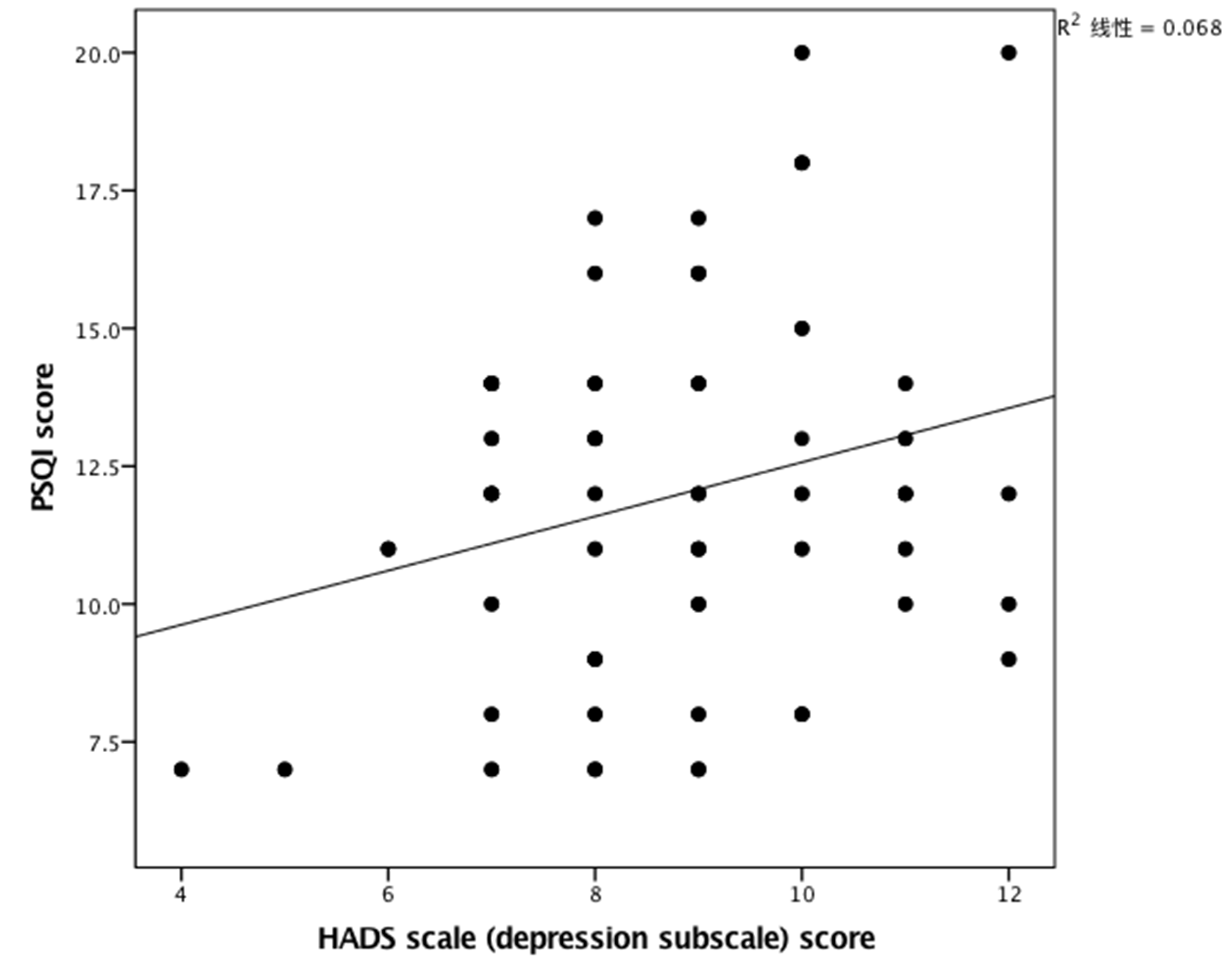

Supplement: Supplementary file 3 — Scatter plot of the correlation between sleep quality and depression (PNG 15953 kb) [file 520_2021_6295_Fig3_ESM.png]

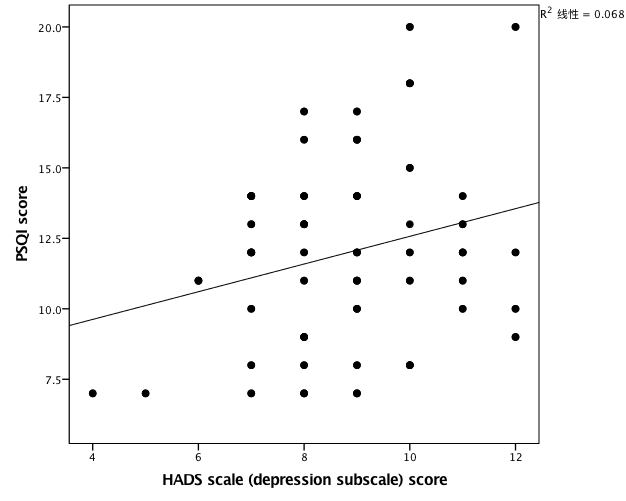

Supplement: Supplementary file 4 — High Resolution Image (TIF 919 kb) [file 520_2021_6295_MOESM2_ESM.tif]

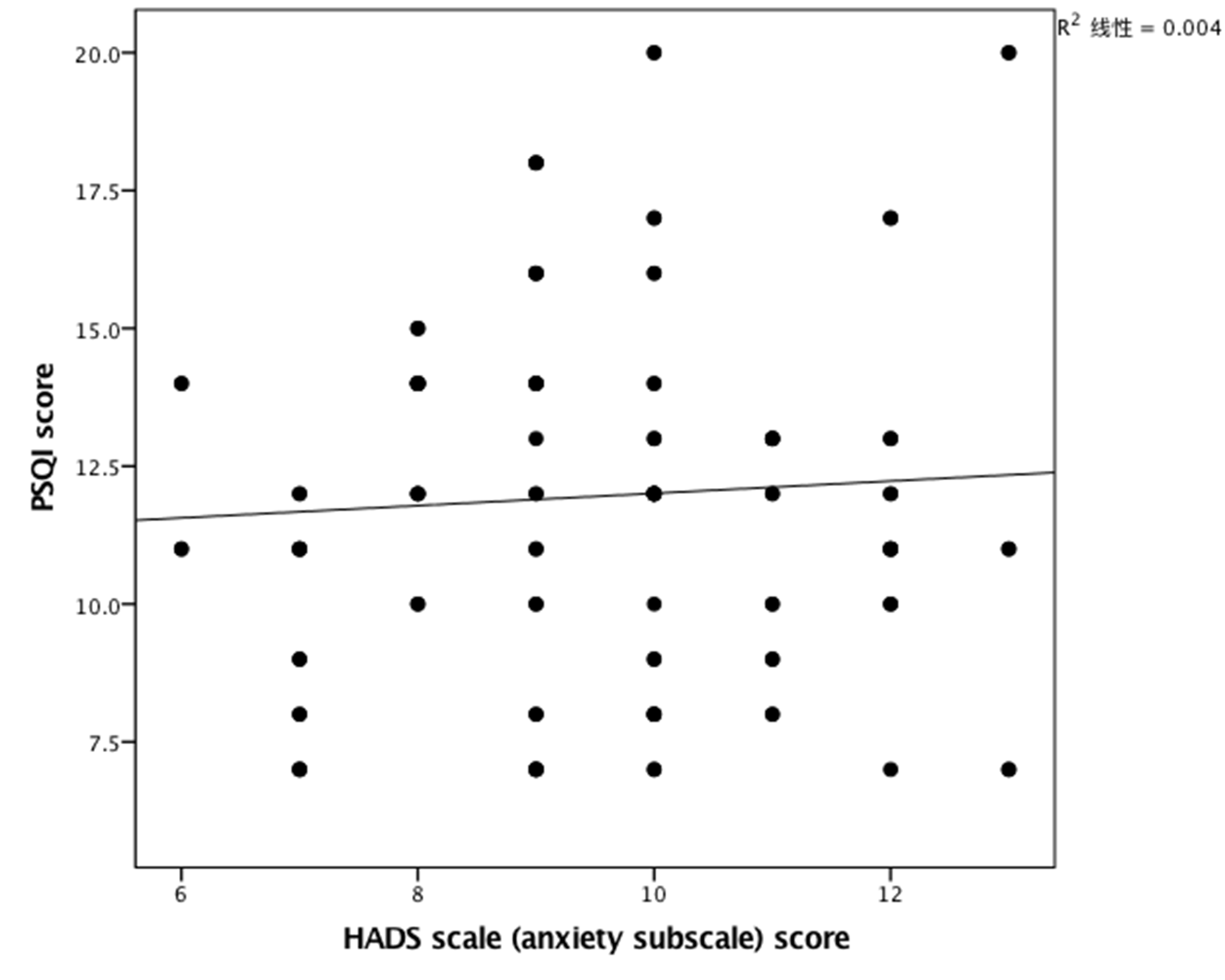

Supplement: Supplementary file 5 — Scatter plot of the correlation between sleep quality and anxiety (PNG 15953 kb) [file 520_2021_6295_Fig4_ESM.png]

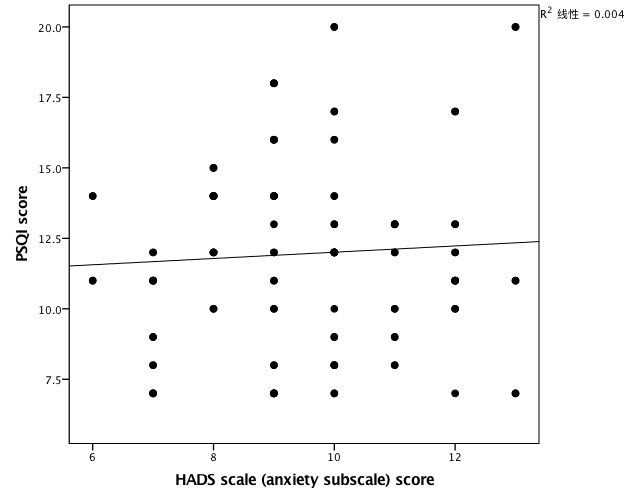

Supplement: Supplementary file 6 — High Resolution Image (TIF 919 kb) [file 520_2021_6295_MOESM3_ESM.tif]
