# Supplementary material for: The characteristics and related factors of insomnia among postoperative patients with gastric cancer: a cross-sectional survey
Source: Support Care Cancer. 2021 May 27;29(12):7315–22. doi: 10.1007/s00520-021-06295-6 (PMC8550093; doi:10.1007/s00520-021-06295-6)
Supplement: Supplementary file 7 — Correlation analysis between chemotherapy cycle and PSQI score (DOCX 14 kb) [file 520_2021_6295_MOESM4_ESM.docx]

| **Supplementary Table 1** Correlation analysis between chemotherapy cycle and PSQI score | | | | | |
| --- | --- | --- | --- | --- | --- |
|  |  | n | PSQI score  M（Q25,Q75） | Z | *P* |
| The number of chemotherapy cycle | ≤6 | 55 | 10 (8, 12) | -4.447 | 0.000^*^ |
|  | ＞6 | 49 | 13 (12, 16) |  |  |
| ^*^*P*＜0.05 | | | | | |
