# Supplementary material for: The characteristics and related factors of insomnia among postoperative patients with gastric cancer: a cross-sectional survey
Source: Support Care Cancer. 2021 May 27;29(12):7315–22. doi: 10.1007/s00520-021-06295-6 (PMC8550093; doi:10.1007/s00520-021-06295-6)
Supplement: Supplementary file 8 — Risk factors (DOCX 14 kb) [file 520_2021_6295_MOESM5_ESM.docx]

| **Supplementary Table 2** Risk factors | | | |
| --- | --- | --- | --- |
|  |  | OR（95% CI） | *P* |
| the number of chemotherapy cycles | ≤6 | 1.00 |  |
|  | ＞6 | 3.640 (1.416-9.357) | 0.007^△^ |
| PFS-CV scale | ≤4 | 1.00 |  |
|  | ＞4 | 4.390 (1.843-10.460) | 0.001^△^ |
| ^△^*P*＜0.05 | | | |
